# Rate and causes of inappropriate stays and the resulting financial burden in a single specialty burns hospital

**DOI:** 10.1186/s12913-022-08772-y

**Published:** 2022-12-17

**Authors:** Nasrin Moradi, Kianoosh Moradi, Rafat Bagherzadeh, Aidin Aryankhesal

**Affiliations:** 1 Department of Health Services Management, School of Health Management and Information Sciences, Iran University of Medical Sciences, Tehran, Iran; 2 Abu Ali Sina Organ Transplant Hospital, Shiraz University of Medical Sciences, Shiraz, Iran; 3 School of Health Management and Information Sciences, Iran University of Medical Sciences, Tehran, Iran; 4 Department of Health Services Management, School of Health Management and Information Sciences, Iran University of Medical Sciences, Tehran, Iran

**Keywords:** Inappropriate admission, Inappropriate stays, Inappropriate hospitalization, Financial burden, Burns patients, Burns hospital

## Abstract

**Background:**

One of the most important challenges facing hospitals is inappropriate admissions and stays the reduction of which can contribute to a decline in healthcare costs without reducing the quality of services. The aim of this study was to estimate the rate and causes of inappropriate stays and their financial burden in a single specialty burns hospital.

**Methods:**

This is mixed methods study conducted in 2021. In the quantitative phase, all medical records of patients admitted to a burn hospital were reviewed and 260 cases were randomly selected. The records were evaluated based on the Appropriateness Evaluation Protocol to estimate the rate and preliminary causes of inappropriate stays and their direct costs. Frequencies and logistic regression were used for the rates and the influential factors in causing inappropriate stay, respectively. In the qualitative phase, 13 senior and middle managers of the hospital were interviewed for their interpretation of the quantitative data and the main causes of inappropriate stays. Qualitative data were analyzed by using Graneheim-Lundman method.

**Results:**

About 28.5% of the patients had at least 1 day of inappropriate stay and about 6% of the total hospitalization days were inappropriate. Marital status, insurance status, and the length of stay were significantly associated with inappropriate admission (*p* < 0.05). In addition, the annual inappropriate admission days and the direct cost imposed on the patients were estimated at 1490 days and $ 66,848.17. The main causes of inappropriate stays are categorized under themes of healthcare providers, service recipients, financial issues, extra-organizational features, and equipment.

**Conclusion:**

A significant percentage of patients experience inappropriate admissions. The number of inappropriate stays, which imposes a high cost on patients, can be reduced by considering the standard criteria for appropriate admissions. In addition, hospital officials can prevent inappropriate stays as much as possible and reduce the costs and increase the productivity of hospitals through proper management and planning as well as a regular monitoring of physicians and patients.

**Supplementary Information:**

The online version contains supplementary material available at 10.1186/s12913-022-08772-y.

## Background

Countries such as Iran, where the total share of health cost is 5–7% of their GDP, need to pay more attention to the efficiency and productivity [1–3]. The big bulk of such increasing costs (tripled during recent years) is related to hospitals that consume 80% of the GDP [4]. There is no gatekeeping system in Iran [5]; therefore, hospital resources should be managed strictly through evaluating the extent of their inappropriate utilization without compromising the quality of health services [6]. According to the studies, inappropriate use of hospital services, especially in long-stay wards, is widespread among countries [1, 7, 8].

Inappropriate admission is considered as one of the weaknesses of healthcare systems even in developed countries and leads to the overuse of health services, such as human resources, beds, medicine, and health funds [9]. Furthermore, it increases hospital costs and reduces the availability of services to patients in critical conditions, which can finally result in poor care and increased mortality and infection rates [10].

Burn care is one of the costliest cares since people of all ages are susceptible to burn injuries [11] which lead to longer hospital stays and impose economic burden on patients, families, community, and government [12, 13]. The annual cost of treating and caring for a burn patient is estimated to be $ 99,773 in Spain and four times the cost of other injuries in Bangladesh [14, 15]. Iran is a country with more than 30 provinces and over 85 million people; however, there are only 20 specialized burn centers across the country [16]. The current study was conducted in Tehran and the only burn hospital which is also a national referral for burn patients, with more than 3000 hospitalized patients and tens of thousands of outpatients a year. Inappropriate use of hospitals beds and unnecessary admissions have been informally reported or observed by the researchers, especially due to lack of a gatekeeping mechanism in the Iranian health system, which lead to a long waiting list for the hospital [17]. Consequently, the assessment and analysis of the use of hospital services can help health policymakers and managers make optimal use of hospital resources and beds. This study, therefore, aimed to estimate the rate of inappropriate stays and identify their causes, estimate the direct cost of such admissions, and then identify the main causes of such admissions from the perspective of the hospital staff.

## Methods

### Study design

The present mixed method study was conducted as sequential research (quantitative-qualitative) in a single-specialty burn hospital affiliated to Iran University of Medical Sciences in 2021. The first phase included a quantitative study to estimate the rate of inappropriate stays, their causes based on the existing protocol, and finally the direct cost of such admissions. The second phase was a qualitative study which was based on participants’ viewpoints inappropriate hospitalization and their interpretation of the findings from the quantitative phase.

### Study sample

The quantitative part of the study was conducted on the medical records of patients admitted to a single specialty burns hospital. Morgan Table was used to determine the sample size [18]. The total number of hospitalizations in the hospital was about 3800 in 2019; therefore, a total of 260 cases were determined as the sample. A random sample of patients’ records was selected from gynecology ward (*n* = 51), burn intensive care unit (*n* = 59), pediatric ward (*n* = 16), men’s ward I (*n* = 50), and men’s ward II (*n* = 40), clinic hospitalization (*n* = 25), and healing ward (*n* = 19). The main criterion for the inclusion of patients’ records in the study was days of hospitalization (at least 1 day). The selected records were reviewed to identify inappropriate stays, their causes, as well as the direct costs of such admissions.

The participants of the second (qualitative) phase were nurses and physicians with at least 1 year of work experience in managerial positions, such as hospital Chief Executive Officer (CEO), administrator, chief financial officer, hospital matron, chief nursing director, and head physicians in charge of hospital wards. Hence a total of 13 semi-structured interviews were conducted. The participation in the interview was on a voluntary basis.

### Data collection

The data collection tool at the quantitative phase was the Appropriateness Evaluation Protocol (AEP) the validity and reliability of which has been confirmed in previous studies [19, 20]. The protocol was issued in the United States in 1981 and contains clinical criteria. If the patient’s condition meets at least one of 27 listed criteria (Appendix 1), his/her admission and day of stay is considered appropriate [20].

Healthcare managers usually use AEP and its adaptations to calculate all costs and burdens imposed by inappropriate stays [21]. The protocol is comprised of a set of objective criteria to evaluate the appropriateness of admission and subsequently the days of patient stay [22]. Furthermore, it can justify the level of care provided to the patient based on the presence or absence of these criteria [23].

Two of the researchers, NM and KM, explored patients’ records. They extracted demographic information (that is record code, age, sex, place of residence, ward, diagnosis, insurance, dates of hospitalization and discharge, treating physician specialty, and treatment type) based on which each day of hospitalization was determined as appropriate or inappropriate.

In the qualitative phase, the data were collected through semi-structured interviews. The questions probed into participants’ ideas about statistics on hospital inpatient stays (extracted from the quantitative phase), and the main causes of such inappropriate stays. The interviews were held in one-to-one face to face sessions during which the answers were recorded with informants consent otherwise note taking was done. Prior to the interviews, the topic guide was provided to the interviewees in person or electronically. Then, the interviews were conducted in person and at their workplaces. Each interview lasted 30 to 45 minutes and was given a code to maintain the confidentiality of the data provided by the participants.

### Data analysis

In the quantitative stage, the collected data were entered into SPSS software and analyzed at two levels of descriptive and analytical statistics. To investigate the relationship between inappropriate admission days and patients’ underlying variables, the independent T-test and ANOVA were used, and to determine the factors affecting inappropriate stay, the logistic regression test was applied. The variables that entered the analysis included patient’s sex, age, marital status, length of stay, place of residence, insurance status, hospital ward, diagnosis, physician’s specialty and working experience.

In the qualitative stage, right after each interview and in the shortest possible time on the same day, the recorded information was transcribed verbatim. The analysis process also began as soon as the first interview was transcribed. For the first two interviews, two study members, AA and NM, agreed on the important paragraphs and meaning units related to the main causes of inappropriate stays and started coding. Then, NM conducted all interview analysis based on the framework developed through the initial coding to address the main causes of inappropriate hospitalizations. All themes were refined and agreed by all researchers at the end of the analysis. Coding was facilitated by using MAXQDA 2020 software.

To determine the direct costs imposed on patients due to inappropriate hospitalization, their medical records were reviewed and their hospitalization status in terms of appropriateness was determined. Then, the patients’ inappropriate hospitalization days and the services provided on those days were determined. The costs of visit, bed with an extra 6% of nursing care, consultation, patients’ companions, medicine, and equipment were all calculated according to the tariffs announced by the Ministry of Health in 2019, taken from the accounting department. Then, based on the direct costs imposed on patients (extracted by reviewing their files), the cost per day and the annual cost of inappropriate hospitalizations were estimated. Rials were converted into dollars according to the mean exchange rate by the Central Bank issued in 2019 (US$ 1 = 42,000 Rials). The discount rate was not applied because the analysis horizon was considered within 1 year.

## Results

In this section the findings are provided for both stages of the study, including (i) the quantitative phase: the rate of inappropriate stays, preliminary causes of inappropriate stays, influential factors, and the cost of inappropriate stays, and (ii) the qualitative phase: the main causes of inappropriate hospitalisation.

### Quantitative phase

As shown in Tables 1, 260 cases of hospitalization were reviewed. The mean age of the patients was 37 ± 20 years, about 66.2% of the patients were male, and 65% were married. The mean length of stay for the patients was 6.41 ± 5.84 days.

**Table 1:** Characteristics of selected patient recrods for the quantitative phase of study

Variable	Subgategory	Frequency of underlying variable (%) n
Sex	Male	(66.2) 172
	Female	(33.8) 88
Marital Status	Single	(35) 91
	Married	(65) 169
Place of Residence	Outside of Tehran	(36.2) 94
	Tehran	(63.8) 166
Ward	Children	(6.2) 16
	Healing	(7.3) 19
	Clinic admission	(9.6) 25
	Men II	(15.4) 40
	Men I	(19.2) 50
	Gynecology	(19.6) 51
	B.ICU	(22.7) 59
Insurance status	Insured	(12.3) 32
	Uninsured	(87.7) 228
Diagnosis	1–10% burn	(38.1) 99
	11–20% burn	(20) 52
	21–30% burn	(6.9) 18
	31–40% burn	(5) 13
	41–50% burn	0
	50% < burn	(6.9) 18
	Healing	(18.8) 49
	Debrid graph	(4.2) 11
Medical specialty	General surgeon	(51.5) 134
	Plastic surgeon	(48.5) 126
Physician’s experience	Up to 4 years	(.8) 2
	5-9 years	(14.6) 38
	10-14 years	(26.5) 69
	15-19 years	(15) 39
	20 or more years	(43.1) 112
Age mean	37.03 ± 20	
Length of stay (days)	6.41 ± 5.84	

#### Estimation of inappropriate stays and the associated factors

It can be seen from the data in Table 2 that 186 (71.5%) stays were appropriate and 74 (28.5%) were inappropriate. Only the two variables of insurance status and marital status had a significant relationship with the days of inappropriate admission; in other words, the mean values of inappropriate stays for married people (0.47 ± 0.77) and those with health insurance (0.44 ± 0.75) were higher than those of the single people and the patients without health insurance.

**Table 2:** Frequency and distribution of inappropriate hospitalization days and the determining factors

Variable	Subgategory	P-value1	P-value2	OR (95% CI)	Inappropriate stays (%) cases	Inappropriate stays days* Mean ± SD
Sex	Male *	.16	.7	1.18(.5–2.8)	(26.7)46	.34 ± .63
	Female				(31.8)28	.49 ± .85
Marital Status	Single *	.007	.03	2.65(1.07–6.57)	(19.8)18	.24 ± .58
	Married				(33.1)56	.47 ± .77
Place of residence	Outside of Tehran	.16	.32	1.38(.73–2.63)	(34)32	.48 ± .78
	Tehran *				(25.3)42	.34 ± .67
Ward	Children *		.72	1.6(.36–7.25)	(31.3)5	.25 ± .45
	Healing		.85	1.14(.27–4.82)	(21.1)4	.21 ± .42
	Clinic admission	.54	.46	.6(.14–2.44)	(16.4)	.2 ± .5
	Men II		.47	1.47(.51–4.24)	(30)12	.43 ± .71
	Men I		.6	1.83(.68–4.88)	(36)18	.5 ± .78
	Gynecology		.53	1.32(.46–3.83)	(33.3)17	.43 ± .7
	*BICU		–	–	(23.7)14	.42 ± .87
Insurance status	Insurance	. > 001	.008	8.63(1.77–42.05)	(6.3)2	.06 ± .25
	Uninsurance				(31.6)72	.44 ± .75
Diagnosis	1–10% burn*		–	–	(27.3)27	41 ± .8
	11–20% burn		.62	.97(.4–2.32)	(26.9)14	.33 ± .58
	21–30% burn		.61	1.3(.4–4.37)	(38.9)7	.44 ± .7
	31–40% burn	.95	.88	.8(.17–3.83)	(30.8)4	.54 ± .87
	41–50% burn		0	0		0
	50% < burn		.56	1.31(.38–4.43)	(33.3)6	.44 ± .78
	Healing		.88	1.1(.41–2.52)	(26.5)13	.37 ± .66
	Debrid graph		.63	1.51(.27–8.48)	(27.3)3	.27 ± .46
Physician’s specialty	General surgeon	.14	.73	1.12(.59–2.1)	(306)41	.46 ± .81
	Plastic surgeon*				(26.2)33	.33 ± .6
Physician’s experience	Up to 4 years	.45	.33	4.56(.22–96.28)	(50)1	1 ± 1.14
	5-9 years		.38	4.16(.17–99.42)	(31.6)12	.42 ± .72
	10-14 years		.47	3.07(.14–66.79)	(30.4)21	.41 ± .71
	15-19 years		.42	3.61(.16–82.3)	(28.2)11	.51 ± .97
	20 or more years*		–	–	(25.9)29	.32 ± .6
Patient age				.19	.98(.96–1.04)	
Length of stay				<.001	1.11(1.05–1.17)	

*reference group* 1. Independent T-test and ANOVA outcomes 2. Logistic regression outcomes

In order to determine the factors affecting the inappropriate stay of the patients, the logistic regression test was used, and it was found that three variables, that is, marital status, insurance status, and length of stay influenced inappropriate stays. Accordingly, married patients were about 2.65 times more likely to be inappropriately hospitalized than single ones. In addition, the patients living outside Tehran were 1.38 times more likely to be inappropriately hospitalized than the patients in Tehran, and those with health insurance were about 8.63 times more likely to be admitted inappropriately than the patients without health insurance.

The length of stay also had a direct effect on the likelihood of inappropriate stay, meaning that in case of adding one unit to the length of a patient’s hospital stay, the risk of inappropriate stay would increase by 1.11 times.

General surgeons were 1.12 times more likely to prescribe inappropriate hospitalizations than plastic surgeons. Considering the physician’s work experience variable, patients treated by lower-experienced physicians were more exposed to inappropriate hospitalization than those treated by higher-experienced physicians. In other words, inappropriate stays for patients treated by physicians with 2 or 3 years of experience was 4.56 times more than those treated by physicians with 20 years of experience.

#### Reasons for inappropriate stays

As presented in Tables 3, 40 days of the total inappropriate stays (*N* = 102) were due to lack of patient visits (39.21%) followed by physicians’ conservatism and the problems with settlement and discharge from hospital. Thus, the total number of inappropriate hospitalization days was estimated at 1490 days.

**Table 3:** Causes of inappropriate hospitalization days in the medical records of the study participants based on the AEP

Causes of inappropriate hospitalization days	Number of days	Percent
Waiting for test results	8	7.84
Postponed surgery	9	8.82
No consultation by the physician	11	10.78
No visit by the physician	40	39.21
Conservatism and other physician reasons	21	20.6
Discharge and settelment problems	13	12.75
Total number	102	100
Annual estimate	1490	

#### Direct cost of inapposite stays

The total direct costs imposed on the patients in 102 days of inappropriate hospitalization was $ 4576. 18, that is $ 44.86 a day (Table 4). Thus, the total direct costs imposed on the patients for 1490 days of inappropriate hospitalization was estimated at $ 66,848.17.

**Table 4:** Total direct costs of inappropriate hospitalization days

Cost item	Total cost of inappropriate admissions (USD)	Cost of inappropriate admissions per day (USD)	Estimation of annual inappropriate admissions cost (USD)
Bed	2095.56	20.54	30,612.97
Physician visits	199.07	1.95	2908.01
Consultatioins	38.08	.37	556.26
Patient’s companion	998.14	9.78	14,580.71
Medication	510.71	5	7460.43
Diagnostic tests	127.38	.12	1860.72
Meals	607.14	5.95	8869.04
Total direct cost	4576.18	44.86	66,848.17

### Qualitative phase

Table 5 summarizes the groups and subgroups of the main causes of inappropriate hospitalization, such as clinicians, patients, financial issues, extra-organizational issues, and equipment.

**Table 5:** Root causes of inappropriate hospitalization days from the perspective of physicians and nurses

Group	Clinicians	Patients	Financial problems	Extra-organizational	Equipment
**Subgroup**	• Induced demand by physician • Lack of daily presence of doctor • Lack of resident orthopedist • Wrong diagnosis • Physicians’ conservatism • Lack of timely counseling • Surgery cancellation • Busy operating room	• Long distance of patients from outside of Tehran • Insistence of the patient and his/her family • Elderly patients and those with underlying disease • Homeless or unsupervised people • Family-less people • Absence of patient’s companion • Patients of other nationalities • Lack of accommodation facilities • Patient’s fear of the wound getting worse • Patient reluctance to surgery • Lack of patient cooperation in treatment or care	• Patients’ lack of insurance coverage • Induced demand of patients with supplemental insurance • High treatment cost of burn patients • Patient’s unfavorable economic status • Problems with the settlement of homeless, unsupervised, and family-less patients	• Lack of cooperation of interested organizations in admitting patients with wounds • Lack of inter-hospital cooperation • Lack of cooperation of patient’s family in taking care of patients • Road closures due to corona restrictions	• Lack of orthopedic equipment • Lack of ultrasound equipment

#### Clinicians

Certain admissions occurred due to induced demands by physicians, misdiagnosis, lack of daily rounds by physicians, absence of resident orthopedists, physicians’ conservatism, lack of timely consultation, surgery cancellation, and busy operating room.

“*Some percent of a patient’s hand skin is burnt. Some surgeons tell the patient to go home to get better, but some others, tell him to come and have an unnecessary surgery.*” (Participant 1).

“*Physicians sometimes misdiagnose grade-one burn by higher grades. This cause inappropriate hospitalizations.*” (Participant 6).

“*We have a lot of patients with electrocution, most of whom need amputation. The orthopedist only comes once a week.*” (Participant5).

“*Some physicians are afraid to discharge the patients with wounds without indication and try to keep them in the hospital conservatively*” (participant 8).

#### Patients

According to the participants one of most influential factors affecting inappropriate hospitalization days was related to patients’ insistence to stay in hospital, especially those living outside of Tehran, foreigners, homeless or unsupervised patients, and the family-less ones without accommodation. In other occasions, lack of patients’ cooperation during the process of treatment, their fear of wound worsening, or unwillingness to undergo surgery were the reasons for inappropriate stays.

*“The patient and his companion insisted to be hospitalized as they had come from far away and did not have accommodation*.” (participant1).

“*Some patients don’t leave the hospital for fear of their wounds get worse.*” (Participant7).

#### Financial issues

One of the causes of inappropriate stays at the hospital was financial problems. The high cost of treating burn patients, their poor economic status, and lack of insurance coverage had led to settlement problems.

“*One of the patients had been treated for 80 million Tomans ($ 2666.6) because he had 5 or 6 surgeries, when he was discharged, he stayed in the hospital for an extra month because he didn’t have insurance coverage or money to pay*” (Participant 11).

#### Extra-organizational issues

Hospitalization is influenced by the cooperation of other organizations. Non-cooperation of organizations, such as prisons, welfare, and social emergencies in admitting patients with wounds, lack of inter-hospital cooperation, lack of cooperation of patients’ families in taking care of patients and closure of roads due to corona restrictions were extra-organizational issues that had caused longer inappropriate hospitalizations.

“*We had a veteran patient with mental problem, and his family and supporting foundations weren’t willing to accept him. He remained in the hospital for about two extra weeks.*” (Participant 4).

#### Equipment

Lack of diagnostic and therapeutic equipment was recognized as another factor causing inappropriate stay and affected the quality hospitals’ performance.

“*There is no ultrasound equipment here, so we often have to transfer patients out of the hospital which may increase the length of hospitalization*” (participant 9).

## Discussion

Appropriate hospitalization is considered as a monitoring tool and an instrument for the evaluation of resource allocation in hospitals and has an important impact on the efficiency of healthcare systems. The aim of this mixed method study was to identify the causes of inappropriate says and to calculate the resulting financial burden in a specialized burn hospital for the first time in Iran.

In this study, the status of inappropriate hospitalizations was evaluated by the use of AEP. The results indicated that about 6% of hospitalization days were inappropriate. This is in line with the results of the studies by Lee et al. [24], Mannocci et al. [25], Aledo [26], Tavakoli et al. [27], Meidani et al. [28], and Nekooei Moghadam et al. [29] who found inappropriate stays in 23.24, 22, 24.6, 20, 6.3, and 9% of cases, respectively. It seems that these different ranges of inappropriate stays depended on the type of hospitals studied, hospital wards, and demographic information, such as age, sex, insurance status, and length of hospital stay.

The results of the present study indicated that only two variables, that is insurance status and marital status had a significant relationship with inappropriate hospitalization days. The mean number of inappropriate hospitalization days was higher among married patients and those with health insurance. In other words, the patients who had insurance were about 8.63 times more likely to be hospitalized inappropriately, this is consistent with the studies by Barouni et al. and Fekari et al., who observed a statistically significant difference between inappropriate hospitalization and insurance [30, 31]. However, Soltani’s study showed that the percentage of inappropriate stays was higher in uninsured people [32]. It seems that uninsured patients with Low burn level, who had to pay all hospital costs, had the shortest stay in the hospital, whereas having insurance affected their length of inappropriate stay.

The present study indicated that, married patients were approximately 2.65 times more likely to be hospitalized inappropriately than single patients. In a systematic review, Kouhestani et al. showed that the percentage of inappropriate stays was higher among married people (16.1%) than single ones (10.9%) [33]. It is likely that married people are older than single ones; therefore, the older the patients are, the more they use health services.

In this study, length of stay also had a direct effect on the probability of inappropriate stay. In other words, in case of adding one unit to the length of a patient’s hospital stay, the risk of inappropriate stay would increase by 1.11 times.

The most important causes of inappropriate stays according to the evaluation protocol of the patients’ files were related to non-daily visits of patients (39.21%), conservatism and other reasons of physicians (20.6%), settlement problems (12.75%), delayed consultation (10.78%), waiting for and postponing surgery (8.82%), and waiting for test results (7.84%). In their study, Barisonzo et al. found that conservative performance (39%), the results of diagnostic tests (21%), and counseling services (18%) were the factors affecting inappropriate hospitalization days [34]. Similarly, Meidani et al. and Tavakoli et al. found that the absence of physicians, delayed counseling, and financial inability of the patients as factors affecting inappropriate hospitalization [27, 28].

In the present study, the total direct cost of inappropriate hospitalization days imposed on the patients was 19,219,994 Tomans ($4576.18), and in the study by Maled et al. in Mexico, the mean cost of inappropriate hospitalization days per patient was $3233 [35]. In their study, Aledo et al. studied two groups and found that the cost was $145,573.36 (€147,044) in the control group and $65,797.38 (€66,462) in the intervention group [26].

In this study, the most important factors causing inappropriate stays from the perspective of physicians and nurses were related to the service providers. These results corroborate with those of the study by Ghods et al., Lee et al., and Campbell et al. who found factors related to health providers as the main motivators for inappropriate hospitalizations [24, 36, 37].

Physicians’ conservatism on patient management, lack of daily presence of surgeons, and the cancellation or postponement of surgery by hospital was mentioned as the causes of inappropriate admissions by most participants. Any improper use of operating rooms could lead to delays in providing care to the patients and as a result, imposes costs on healthcare institutions. According to Kouhestani, physician-related factors accounted for most inappropriate hospitalizations since delays in surgeries or not having access to treating physicians could cause such hospitalizations [33]. Similarly, Ravangard et al. and Mosadeghrad et al. found that Factors causing inappropriate stay include physicians’ specialty, discharge time, rescheduled surgeries, lack of regular attendance of doctors, delay in consultations [38, 39]. Learning about the causes of inappropriate admission can help prevent wastage of resources; moreover, large proportion of financial resources can be saved annually for the health system and the community.

One of the factors involved in inappropriate stays from the perspective of physicians and nurses in this study was service recipients. Most of the participants mentioned factors, such as homeless, unsupervised, or family-less patients, long distances from the patients’ residence to the hospital, and patients from other cities. When the patients who needed daily follow-up dressing services were discharged, they would not leave the hospital due to the lack of accommodation facilities, and this led to inappropriate hospitalizations. It seems that homeless people in Iran do not have a shelter to live to comfort, and these people are often deprived of having and maintaining suitable, safe, secure, and conventional housing. For this reason, they stay in hospital insistently which cause an unnecessary increase in the duration of hospitalization. In line with this finding, Mosadeghrad et al. mentioned patient-related factors [39]; moreover, Massimi et al. and Teke et al. referred to environmental factors, distance from patient’s residence to the hospital, and the need for daily treatment as causes for inappropriate hospitalizations [40, 41].

Based on the results, another factor causing inappropriate stays was the insistence of patients and their families, elderly patients, and the patients with underlying diseases. It seems that some burn patients whose conditions improve after treatment and did not have hospitalization indication did not leave the hospital after discharge, due to the fear of wound worsening and stayed in hospital until the wounds healed completely. This led to inappropriate hospitalizations. Although wound care training was provided to the patients, some of them, due to their psychological conditions, insisted on staying in hospital. Elderly patients and insistence of the patients and their families were identified as inappropriate hospitalization causes in different studies [24, 42–44].

Financial problems were identified as other causes of inappropriate hospitalizations from the perspective of the physicians and nurses in this study. Considering the nature of burn treatment, which usually requires several surgeries, some patients had big problems at the time of discharge due to the lack of insurance coverage which could cause catastrophic costs for them [45]. Some patients inappropriately stayed on hospital beds for a few days after the doctor’s discharge order in order to prepare money for the treatment charges or to seek the social service discounts. It seems that these patients had a high burn level and needed several high-cost surgeries. Besides, some patients with supplemental insurance did not leave the hospital even after being discharged by the doctor because their treatment costs were covered by the supplemental insurance. Financial problems such as insurance and settlement problems were similarly mentioned by Meidani et al. and Tavakoli et al. [27, 28].

This study showed that, extra-organizational issues were also considered as causes of inappropriate hospitalizations from the perspective of the participants. They believed that non-cooperation of the organizations such as prisons, welfare, Martyr Foundation, and social emergency as well as the non-cooperation of other hospitals in continuing supportive treatment of the patients with wounds were the main causes of inappropriate admissions. In a study by Ghods, extra-organizational issues and lack of cooperation among service providers were identified as the reasons for inappropriate admissions [36]. Similarly, Hwang suggested that adequate cooperation in the medical system could reduce the number of inappropriate hospitalizations [46].

Furthermore, tools and equipment were considered as other reasons for inappropriate hospitalizations by the physicians and nurses. Medical equipment is important diagnostic devices; however, due to lack of imaging equipment for the patients in need of ultrasound patients had to spend a lot of time to make an appointment and follow up the diagnostic test results in other centers. This caused delays in the health system and eventually led to inappropriate stays. In line with the results of this study, Kermani et al. referred to the above-mentioned factors as well [47].

One of limitations of this study could be the Covid-19 pandemic conditions and the reluctance of hospital staff to share their experiences due to the sensitive nature of the epidemic. Hence busy staff with higher responsibilities did not attend our study, so the qualitative section can be affected.

## Conclusion

According to the results, a significant percentage of the patients experienced inappropriate hospitalizations elimination of which is very important to achieve an effective healthcare system and optimal use of resources. Having an insurance coverage and being married as well as residing in cities other than the hospital setting can increase the chance of inappropriate admission and stay. Implementation of standard criteria for stays could reduce inappropriate hospitalization days that impose a high cost on patients. Insurance companies can assign more precise view on hospitals’ bills where the inappropriate stays can be deducted from their repayment lists to motivate hospitals for the prevention of inappropriate stays. Therefore, hospital officials can prevent inappropriate stays as much as possible, reduce the costs, and increase hospital productivity through proper management, correct planning, and regular daily monitoring of physicians and patients.

## Supplementary Information

**Additional file 1.**

## Data Availability

All data generated or analyzed during this study are included in this published article [and its supplementary information files].
