# Supplementary material for: Rate and causes of inappropriate stays and the resulting financial burden in a single specialty burns hospital
Source: BMC Health Serv Res. 2022 Dec 17;22:1538. doi: 10.1186/s12913-022-08772-y (PMC9758030; doi:10.1186/s12913-022-08772-y)
Supplement: Supplementary file 1 — Additional file 1. [file 12913_2022_8772_MOESM1_ESM.doc]

Part One: quantitative part

- demographic information

| ID |  | Diagnosis |  |
| --- | --- | --- | --- |
| age |  | Physician’s experience |  |
| gender |  | Medical specialty |  |
| Place of Residence |  | Length of stay |  |
| Unit name |  |  |  |
| Insurance status |  |

Appropriate inpatient assessment criteria

| criteria | yes | No |
| --- | --- | --- |
| Perform surgery on the same day |  |  |
| Schedule surgery the next day to require preoperative counseling or testing |  |  |
| Cardiac catheterization on the same day |  |  |
| Angiography on the same day |  |  |
| Biopsy of one of the internal organs on the same day |  |  |
| Perform thoracentesis paracentesis on the same day |  |  |
| Perform CNG diagnostic tests on the same day |  |  |
| Perform any test that requires strict diet control during the test |  |  |
| Perform new or experimental therapies that require patient monitoring or dose adjustment |  |  |
| Record information about the patient's clinical condition by the physician at least 3 times a day |  |  |
| The day after surgery or diagnostic procedures mentioned in numbers one and numbers 3 to 7 |  |  |
| Respiratory care at least 3 times a day |  |  |
| Intravenous injections of fluids, electrolytes, drugs, proteins intermittently or permanently on that day |  |  |
| Monitor vital signs continuously for at least 30 minutes every 4 hours of the day |  |  |
| Intramuscular or subcutaneous injections at least twice a day |  |  |
| out put. Intake measurement that day |  |  |
| Supportive measures such as emptying cavities (tube and chest tube) and large surgical wounds on that day |  |  |
| Record clinical information by nurses with a doctor's order at least 3 times a day |  |  |
| Inability to defecate or not empty the bowels in the last 24 hours so that it can not be attributed to neurological disorders |  |  |
| Blood transfusion within 48 hours to compensate for lost blood |  |  |
| Acute myocardial ischemia seen on ECG or ventricular fibrillation in the last 48 hours |  |  |
| Fever above 37.8 in the last 48 hours |  |  |
| Coma - Failure to respond to stimulation for at least one hour during the past 48 hours |  |  |
| Sudden and acute decrease in the level of consciousness in the last 48 hours (except in cases of alcohol deprivation) |  |  |
| Sudden and acute disorder of blood cells; Neutropenia, Anemia, Thrombocytopenia, Leukocytosis, Erythrocytosis Thrombocytosis within 48 hours |  |  |
| Acute and progressive disorders of the nervous system during 14 days |  |  |
| Occurrence of a newly proven MI or CVA during |  |  |

Qualitative part

demographic information

| Code of Interviewed nurse |  | Code of the interviewed physician |  |
| --- | --- | --- | --- |
| age |  | age |  |
| gender |  | gender |  |
| Level of education |  | Type of specialty of the treating physician |  |
| Unit name |  | Unit name |  |
| Duration of work experience |  | Duration of work experience |  |

What is the hospitalization status?-

What do you think are the causes of inappropriate hospitalization?-

What causes an inappropriate hospitalization in your opinion?-

? How have you had any problems with inappropriate hospitalization-
